# Experimental and numerical investigation of the mechanical performance of natural–synthetic hybrid composite laminates

**DOI:** 10.1371/journal.pone.0354203

**Published:** 2026-07-17

**Authors:** Shaik Irfan Sadaq, Dakuri Vasudev Srividya, Abhishek Agarwal, Balram Yelamasetti, Samera Saniya, Jamyang Choden

**Affiliations:** 1 Department of Mechanical Engineering, Muffakham Jah College of Engineering and Technology, Hyderabad, Telangana, India; 2 Department of Mechanical Engineering, University College of Engineering, Osmania University, Hyderabad, Telangana, India; 3 Department of Energy Technology, Tallinn University of Technology, Tallinn, Estonia; 4 Department of Mechanical Engineering, Sreyas Institute of Engineering and Technology, Hyderabad, Telangana, India; 5 Department of Physics, Muffakham Jah College of Engineering and Technology, Hyderabad, Telangana, India; 6 Department of Mechanical Engineering, College of Science and Technology, Royal University of Bhutan, Phuentsholing, Bhutan; King Mongkut’s University of Technology North Bangkok, THAILAND

## Abstract

Fiber-reinforced polymer composites have attracted considerable attention in engineering applications owing to their high strength-to-weight ratio, corrosion resistance, and tailorable mechanical properties. Hybridization provides an effective approach for combining the advantageous characteristics of different reinforcement materials to achieve improved overall laminate performance. In the present study, three six-ply hybrid composite laminates, namely J2/C2/J2, J2/G2/J2, and C2/G2/C2, with a [0°/90°] stacking sequence and a relatively high fiber content obtained through controlled hand lay-up fabrication, were fabricated using the hand lay-up technique. Test specimens were prepared according to the relevant ASTM standards and subjected to tensile, compressive, and flexural testing. In addition, finite element simulations were performed using ANSYS Mechanical APDL 2020 R1 to validate the experimental observations. The experimental results demonstrated that natural–synthetic fiber hybridization significantly enhanced the mechanical performance of the investigated laminates. The incorporation of carbon fiber into jute-based laminates resulted in an average strength improvement of approximately 88%, whereas glass-fiber incorporation produced an improvement of approximately 55%. Among the investigated configurations, the C2/G2/C2 laminate exhibited the highest tensile strength (approximately 140 MPa), compressive strength (approximately 39 MPa), and flexural strength (approximately 109 MPa). The numerical predictions showed good agreement with the experimental measurements, with maximum deviations below 1.2%. The results confirm the effectiveness of hybridization in enhancing the mechanical response of composite laminates and identify the C2/G2/C2 configuration as the most promising among the investigated laminate systems. These findings demonstrate the influence of natural–synthetic fiber hybridization on laminate mechanical performance and provide useful information for the design of lightweight composite structural components. Further studies involving pressure-vessel fabrication and internal-pressure testing are required before pressure-vessel applications can be established.

## Introduction

Fiber-reinforced polymer composites have gained significant attention in engineering applications owing to their high specific strength, high specific stiffness, corrosion resistance, and design flexibility. Compared with conventional metallic materials, composite laminates offer substantial weight reduction while maintaining desirable mechanical performance. Consequently, composite materials are increasingly utilized in aerospace, automotive, marine, and pressure-vessel applications [1–3]. The most significant and effective ways to fortify and enhance the performance of composite materials is through the hybridization process [4]. Synthetic fibers offer greater rigidity while natural fibers are inexpensive and environmentally good due to their biodegradability. Hybridization of natural and synthetic fibers provides an effective approach for balancing mechanical performance, sustainability, and material cost. Synthetic fibers generally offer high strength, stiffness, and durability, whereas natural fibers are lightweight, renewable, biodegradable, and economically attractive [5]. Hybrid composites combining these reinforcement types can achieve a desirable balance between structural performance and environmental sustainability. By integrating the advantages of both natural and synthetic fibers, hybrid fiber-reinforced composites have emerged as promising materials for a wide range of engineering applications [6,7].

The matrix and reinforcement significantly influence the strength and stiffness of composite materials [8]. The mechanical properties of laminates are determined by variations in composition and layer structure [9,10]. While the elastic modulus is generally unaffected by stacking order, both failure strain and tensile strength tend to increase with the number of plies [11]. Among the factors affecting composite performance, fiber orientation has the greatest effect, followed by the number of laminates and the resin type. Composites with 60 wt.% fiber content exhibited higher mechanical performance than those with 40 wt.% fiber, regardless of layer orientation or number of plies [12,13]. In general, 5-ply composites display slightly better mechanical properties than 3-ply laminates. As fiber volume fraction increases, both stiffness and strength improve, and the damage behavior of the specimens varies accordingly [14,15]. Epoxy composites reinforced with multi-axial, multi-ply fabrics oriented at ±45° and 0/90° demonstrate comparable mechanical performance for the biaxial ±45° and 0/90° laminates [16–18]. For tensile and compressive loading, a fiber orientation of 0° is ideal, as it minimizes deformation and strain, while 0° and 30° angles provide superior strength compared with other orientations [19]. Carbon and glass fiber composites perform best at 0° and 90° [20–22], with unidirectional laminates exhibiting the highest tensile and flexural strengths and moduli [23]. The stacking sequence and ply orientation also influence strain-rate sensitivity; under high strain rates, matrix–fiber boundaries may undergo ductile failure [24]. Hybrid laminates generally outperform non-hybrid composites in tensile, compressive, and flexural tests and are less affected by low temperatures [25,26]. Studies on polymer composites confirm that natural-fiber composites (NFCs) can be comparable or superior to synthetic-fiber composites (SFCs). Jute fiber is an effective reinforcement alternative, and combining it with matrices such as polypropylene, polyester, or epoxy can enhance composite properties. Additionally, chemical treatment of jute fibers can further improve mechanical performance [27,28]. Recent studies have highlighted the growing importance of sustainable composite systems and the development of bio-based composite materials for engineering applications. Bio-based and hybrid composites reinforced with natural and synthetic fibers have demonstrated considerable potential for achieving a balance between mechanical performance, sustainability, and cost effectiveness [29]. Recent investigations have further emphasized circular material pathways, sustainable composite-product development, and the relationship between composite composition and structural performance. In addition, sustainable epoxy composites reinforced with natural and synthetic fibers have demonstrated promising mechanical performance for lightweight structural applications, including automotive body panels and transportation components. These studies collectively indicate that hybridization strategies can provide enhanced structural performance while supporting sustainable material utilization [30–33]. However, direct comparisons among jute/carbon, jute/glass, and carbon/glass laminate systems fabricated under identical processing conditions remain limited.

The least amount of strain is seen in glass/epoxy composite materials oriented at a 90° angle. Comparing its reinforcement to composites made from jute and plain glass weaved the former exhibits significantly better flexural capabilities. An increase in the glass/epoxy composite laminate’s fiber content is allied with a decrease in tensile resistance [34–37]. The flexural, tensile & axial compression strengths of the CF/epoxy resin composite at a 90-degree orientation are superior, and the maximum load was also noted at a high yield point [38] Compared to CF/phenolformalhyde resin composite, the mechanical characteristics of CF/epoxy resin composite are better [39–41]. In longitudinal direction enormous tensile strength of Carbon/epoxy composite laminates are observed and a moderate compressive strength is seen in transverse direction. It was noted that the mechanical performance depended on the fiber’s orientation and thickness. Related to other composites, the reinforced carbon fiber composite has a higher ductility [42]. Glass fiber incorporating with jute fiber composites improves their mechanical qualities and this increases the usage of natural fiber in a variability of applications [43,44]. The flexural test was validated by the hybrid laminate Glass/Jute/Polyester, which displayed the greatest storage modulus values among the jute/polyester and glass/polyester combinations [45].

At equal carbon-fiber and glass-fiber volume fractions (50:50), the maximum hybrid effect was reported. Compared with the corresponding full carbon-fiber and full glass-fiber composites, the hybrid configuration demonstrated improved strength characteristics [46]. The final fracture load causes a dramatic decline in the tensile test results for the glass/epoxy and carbon/epoxy composites, which increase linearly for θ = 90° and non-linearly for θ = 0° till they reach their extreme value [47]. Glass fibers have a far lower modulus than carbon fiber; as the hybrid ratio increases, so do the flexural and tensile moduli. Tensile modulus falls as hybrid ratio rises [48]. The hybrid laminate with the outer carbon fiber layer (CGGC) offers the highest flexural strength [49–51]. The flexural capacity of the composites was enhanced when fibers of carbon were present in the skin areas, while this strength was reduced when glass fibers were present [52]. Compared with carbon composite laminate the hybrid composite laminates are more ductile and it also exhibit more brittle behavior than glass composite laminate [53].

The literature indicates that extensive research has been conducted on conventional composite laminates, whereas comparatively fewer studies have focused on hybrid laminates incorporating both natural and synthetic fibers. Although natural-fiber composites, synthetic-fiber composites, and selected hybrid systems have been widely investigated, a systematic comparison of jute/carbon, jute/glass, and carbon/glass hybrid laminates fabricated under identical processing conditions remains limited. Furthermore, experimental investigations supported by finite element validation are relatively scarce for these material combinations. The [0°/90°] ply orientation was selected because orthogonal fiber arrangements are widely employed in composite structures and have been reported to provide balanced tensile, compressive, and flexural performance while maintaining manufacturing simplicity. This orientation also facilitates effective load transfer along the principal material directions and provides a suitable basis for comparing the influence of different hybrid fiber combinations. The present investigation was limited to the conventional [0°/90°] laminate configuration to ensure consistent comparison among the selected hybrid material systems while minimizing the influence of additional design variables. Alternative laminate architectures, including angle-ply and quasi-isotropic stacking sequences, may exhibit different stress-transfer mechanisms, stiffness characteristics, and failure responses. Therefore, the findings reported in this study should be interpreted within the context of the selected laminate configuration, and future investigations may explore the influence of alternative stacking sequences on hybrid laminate performance.

Although substantial research has been conducted on natural-fiber composites, synthetic-fiber composites, hybrid laminates, and composite pressure-vessel materials, several limitations remain in the current literature. Previous investigations have examined the failure behaviour and structural response of composite pressure vessels reinforced with both synthetic and natural–synthetic fiber systems [21,22]. However, most studies have focused on specific material systems, pressure-vessel geometries, or individual loading conditions. Direct material-level comparisons among jute/carbon, jute/glass, and carbon/glass hybrid laminates fabricated under identical processing conditions and evaluated through combined experimental and numerical approaches remain limited. A concise summary of the major research gaps identified from the literature and the corresponding contribution of the present study is provided in Table 1.

**Table 1:** Research gaps identified in previous studies and contributions of the present work.

Research Area	Findings Reported in Previous Studies	Identified Gap	Contribution of the Present Study
Natural fiber composites	Jute-reinforced composites provide sustainability and reduced cost but generally exhibit lower mechanical performance than synthetic-fiber composites [27,54,55].	Limited structural performance restricts their application in load-bearing components.	Investigates hybridization of jute with carbon and glass fibers to improve mechanical behavior while retaining partial natural-fiber content.
Carbon/glass hybrid laminates	Carbon–glass hybrid laminates exhibit improved tensile and flexural performance compared with single-fiber systems [46,49,52].	Limited comparison with natural–synthetic hybrid laminate architectures under identical fabrication conditions.	Evaluates and directly compares J2/C2/J2, J2/G2/J2, and C2/G2/C2 hybrid laminates manufactured using the same process parameters.
Influence of laminate architecture	Previous studies reported that stacking sequence significantly affects load transfer and failure behavior [23,45].	Insufficient understanding of the influence of specific natural–synthetic hybrid stacking arrangements.	Examines the effect of three different stacking configurations while maintaining identical ply orientation and fiber content.
Experimental characterization	Most reported investigations focus on either tensile or flexural performance individually [36,56].	Comprehensive evaluation of tensile, compressive, and flexural behavior remains limited for the selected hybrid systems.	Provides comparative experimental assessment under tensile, compression, and flexural loading conditions.
Numerical validation	Finite element analysis has been widely used for synthetic-fiber laminates and pressure-vessel structures [53].	Experimental–numerical correlation for jute/carbon, jute/glass, and carbon/glass hybrid laminates remains scarce.	Develops and validates finite element models against experimental results for all investigated laminate configurations.
Pressure-vessel-oriented material selection	Composite pressure-vessel studies mainly focus on fully synthetic reinforcement systems [20–22].	Limited information is available regarding the suitability of natural–synthetic hybrid laminates as candidate materials for future pressure-vessel structures.	Provides preliminary mechanical-performance assessment of hybrid laminates for lightweight structural applications and future composite pressure-vessel-related investigations.

As summarized in Table 1, previous studies have independently investigated natural-fiber composites, synthetic-fiber composites, hybrid laminate architectures, and composite materials for pressure-vessel-related applications. However, a systematic experimental and numerical comparison of J2/C2/J2, J2/G2/J2, and C2/G2/C2 laminate configurations fabricated under identical processing conditions and evaluated under tensile, compressive, and flexural loading conditions remains limited. The selected laminate architectures were intentionally chosen to represent three distinct hybridization strategies. The J2/C2/J2 laminate was selected to evaluate the reinforcing effect of carbon fiber within a natural-fiber-dominant laminate system, whereas J2/G2/J2 was selected to investigate a lower-cost natural–synthetic hybrid alternative incorporating glass fiber. The C2/G2/C2 configuration was included to examine the interaction between two high-performance synthetic reinforcements and to establish a benchmark for comparison with the natural–synthetic hybrid laminates. Maintaining identical ply numbers, fiber orientations, fabrication procedures, and testing conditions enabled direct assessment of the influence of fiber type and stacking arrangement on laminate performance. To address the identified research gap, the present study investigates the mechanical behavior of these hybrid laminate systems through experimental characterization and finite element analysis (FEA). The tensile, compressive, and flexural responses of the laminates are comparatively evaluated to identify the configuration exhibiting the most favorable overall mechanical performance. To the best of the authors’ knowledge, a comprehensive experimental–numerical assessment of these three hybrid laminate configurations under identical manufacturing and testing conditions has not been widely reported in the open literature. The findings contribute to a better understanding of the influence of natural–synthetic fiber hybridization and laminate architecture on the mechanical behavior of hybrid composite systems and provide useful insights for lightweight structural applications.

## Materials and methods

One of the earliest and most widely used methods for fabricating fiber-reinforced polymer (FRP) composites is the hand lay-up technique due to its simplicity, cost-effectiveness, and reliability. The reinforcements used for laminate fabrication consisted of bidirectional woven jute, glass, and carbon fiber fabrics, each having an areal density of 220 g/m^2^. The matrix material comprised epoxy resin used together with the corresponding hardener recommended by the supplier. All reinforcement materials were procured from VRUKSHA Composites, Andhra Pradesh, India.

The constituent materials used for laminate fabrication were characterized based on their physical form, areal density, and thickness. The reinforcing fibers consisted of bidirectional woven jute, glass, and carbon fabrics, while epoxy resin was used as the matrix material. The basic specifications of the reinforcing materials employed in the present investigation are summarized in Table 2.

**Table 2:** Specifications of reinforcing materials used in the fabrication of hybrid composite laminates.

Material	Form	Areal Density (g/m²)	Thickness (mm)	Supplier
Jute Fiber	Bidirectional woven fabric	220	0.65	VRUKSHA COMPOSITES, India
Glass Fiber	Bidirectional woven fabric	220	0.5	VRUKSHA COMPOSITES, India
Carbon Fiber	Bidirectional woven fabric	220	0.5	VRUKSHA COMPOSITES, India

The selected reinforcing fibers exhibit distinct characteristics that make them suitable for hybrid composite applications. Carbon fiber provides high stiffness and strength, glass fiber offers a balanced combination of mechanical performance and cost-effectiveness, whereas jute fiber contributes to reduced material cost and partial replacement of synthetic reinforcement. The combination of these materials enables evaluation of the influence of natural–synthetic fiber hybridization on the mechanical behavior of composite laminates.

### Fabrication of hybrid composite laminates (HCL) using hand layup process

**Fig 1:**
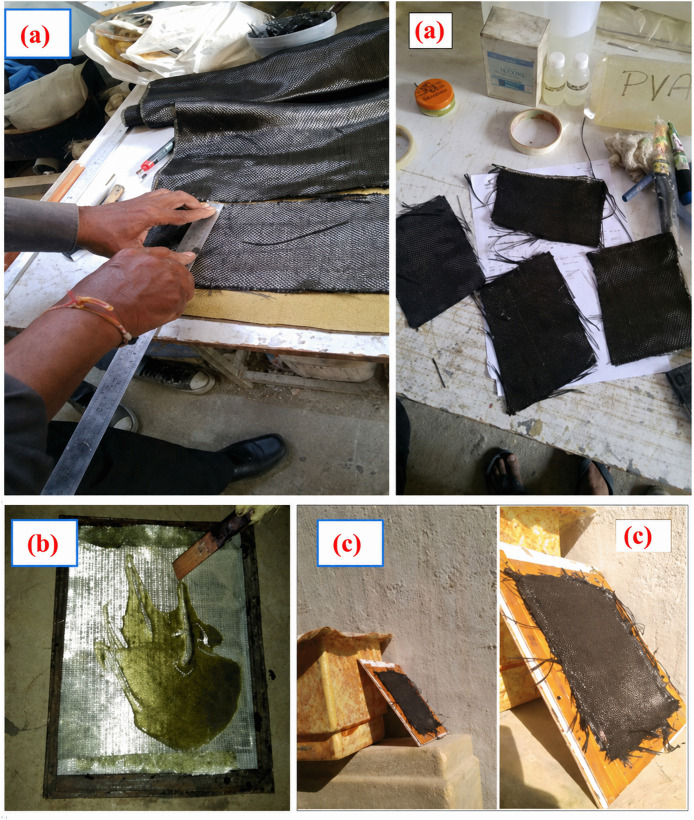
Fabrication stages of the hybrid composite laminates: (a) measurement and cutting of fiber mats, (b) layup and epoxy resin application, and (c) laminate curing process.

**Fig 2:**
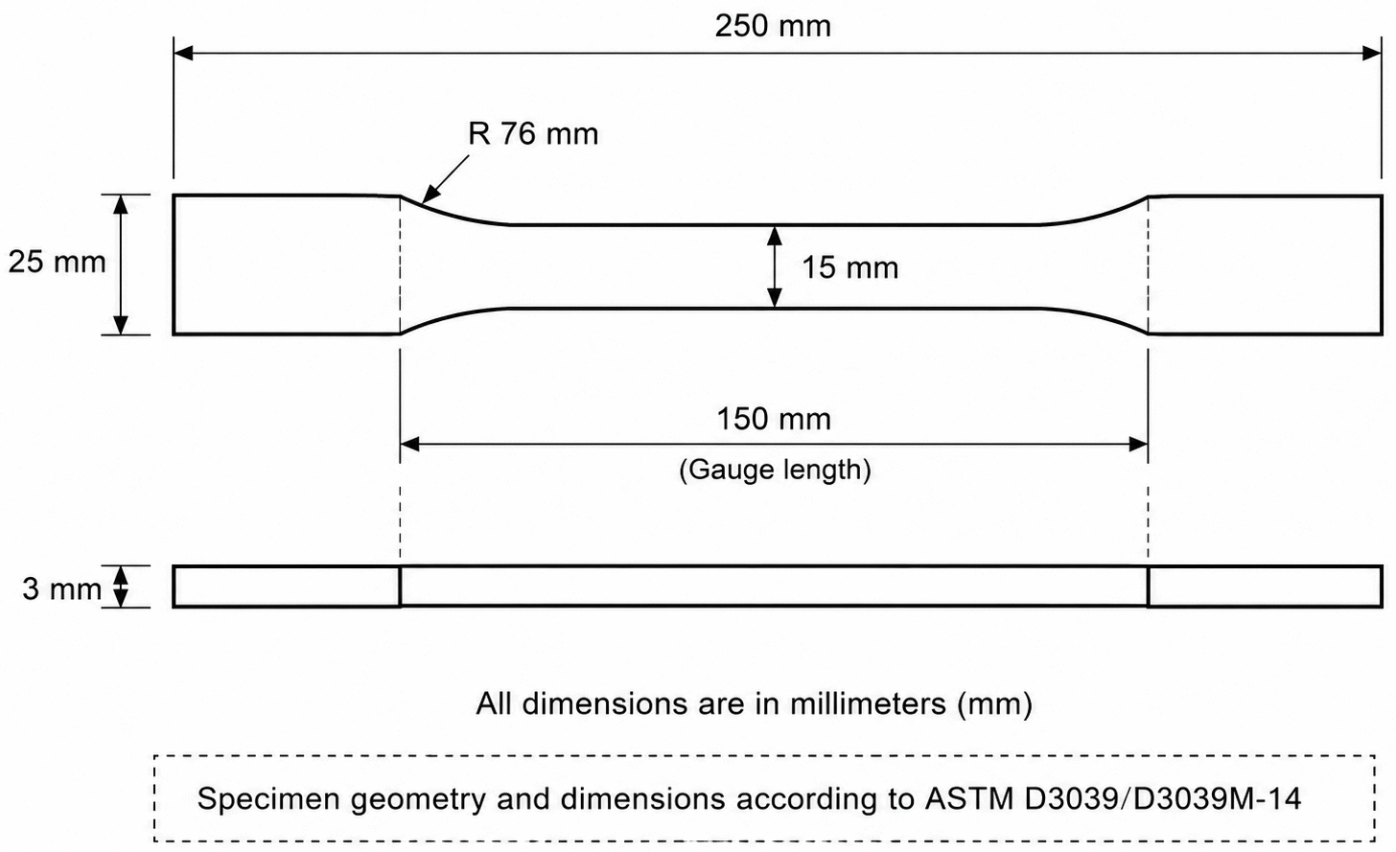
Geometry and dimensions of tensile specimen prepared according to ASTM D3039/D3039M-14.

**Fig 3:**
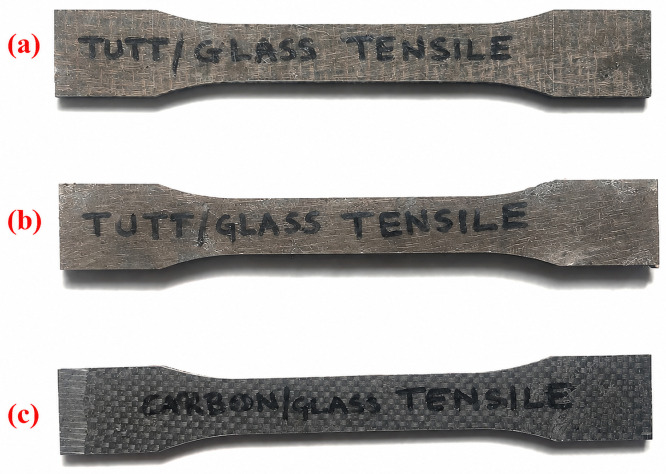
Tensile test specimens of (a) J_2_/C_2_/J_2_, (b) J_2_/G_2_/J_2_, and (c) C_2_/G_2_/C_2._

**Fig 4:**
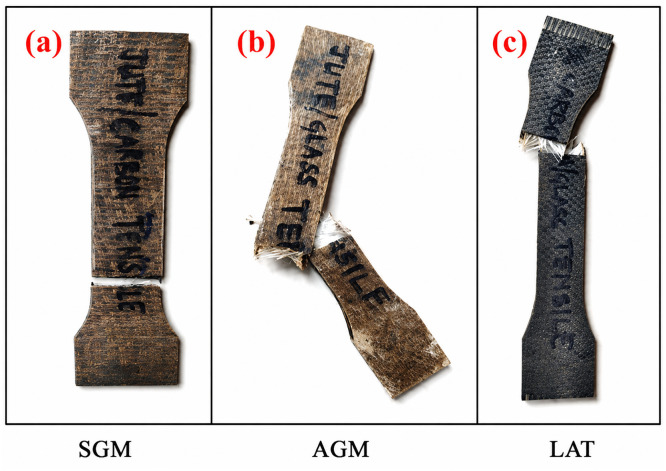
Fractured tensile specimens of (a) J2/C2/J2, (b) J2/G2/J2, and (c) C2/G2/C2 together with the corresponding ASTM D3039 failure codes.

**Fig 5:**
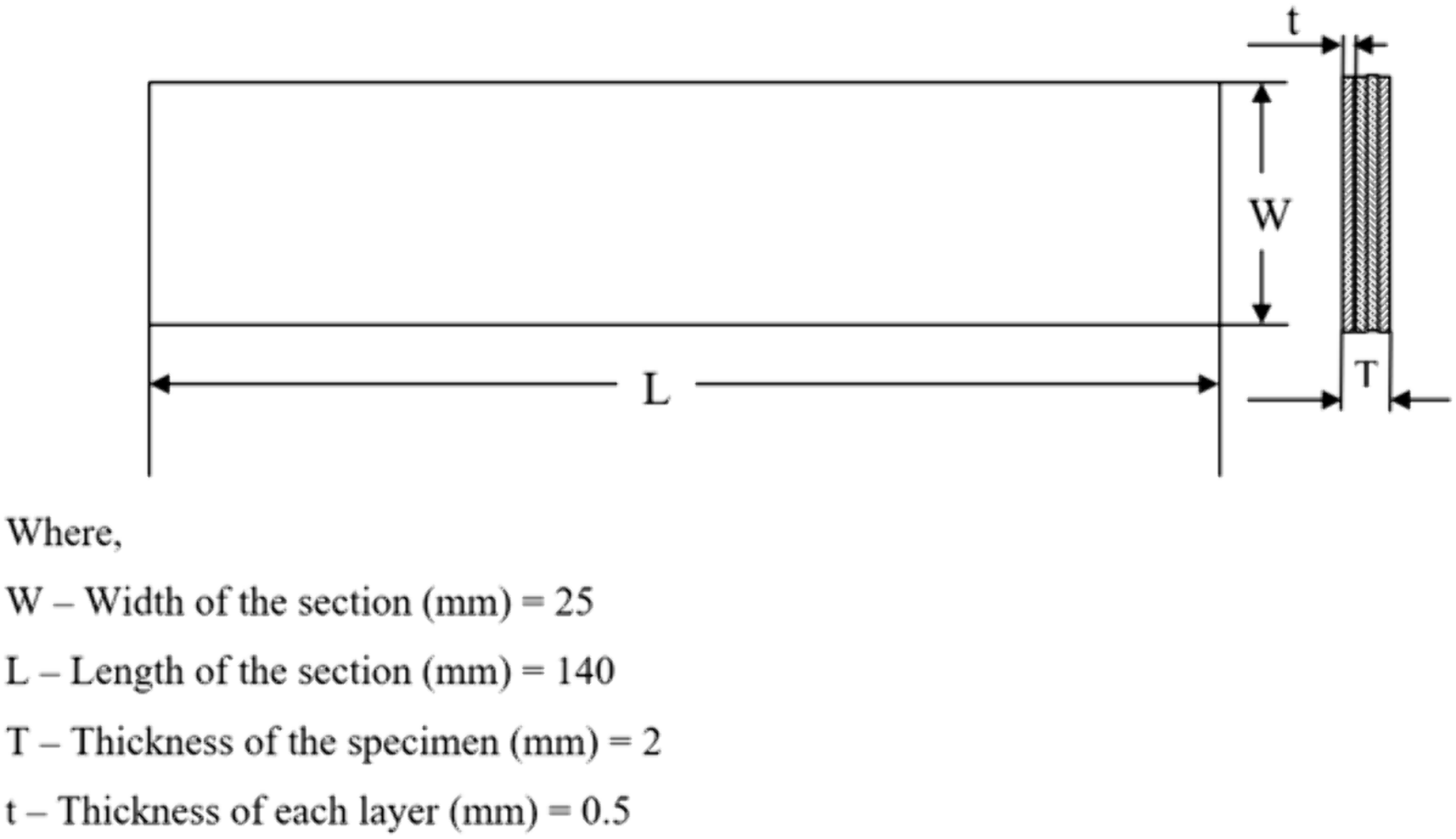
Geometry and dimensions of the compression test specimen according to ASTM D3410/D3410M-16.

All the laminates were produced using the hand lay-up process, as illustrated in Fig 1(a–c), and subsequently machined according to the specimen dimensions specified in ASTM D3039/D3039M-14, ASTM D3410/D3410M-16, and ASTM D790-17 for tensile, compression, and flexural testing, respectively, as shown in Figs 2–5. The laminates were fabricated using controlled quantities of reinforcement and epoxy resin to obtain a relatively high fiber content suitable for structural laminate fabrication. The fiber content was estimated from the constituent material quantities used during manufacturing and was not independently verified through burn-off testing, density measurements, or microscopic image analysis. Therefore, the reported fiber content should be considered an approximate manufacturing estimate rather than a directly measured parameter. Although care was taken to ensure uniform resin distribution and minimize air entrapment during fabrication, void content was not experimentally quantified and therefore was not considered as an independent study parameter. Consequently, the potential influence of manufacturing-induced voids on the mechanical performance of the laminates was not explicitly evaluated and is recognized as a limitation of the present study. Nevertheless, careful resin impregnation, manual rolling, controlled curing, and visual inspection were employed throughout fabrication to minimise air entrapment, resin-rich regions, and fibre misalignment. The thickness of the carbon-fiber and glass-fiber woven fabrics was 0.5 mm, whereas the thickness of the jute-fiber woven fabric was 0.65 mm. The fabrication process involved cutting the reinforcement mats (jute, glass, and carbon woven fabrics) to the mold dimensions of 300 mm × 300 mm, as shown in Fig 1. The epoxy resin was thoroughly mixed with the recommended hardener and then applied between successive fiber layers according to the selected stacking sequences (J2/C2/J2, J2/G2/J2, and C2/G2/C2). The resin was uniformly distributed throughout the laminate thickness to ensure proper impregnation of the reinforcement layers. The fabricated laminates were subsequently cured under ambient conditions, as shown in Fig 1(c).

### Experimental approach of HCL

Since composite laminates consist of reinforcement and matrix materials available in various grades and forms, experimental characterization is essential for accurately determining their mechanical behavior and failure properties. In the present study, three hybrid composite laminate configurations, namely J2/C2/J2 (jute/carbon/epoxy), J2/G2/J2 (jute/glass/epoxy), and C2/G2/C2 (carbon/glass/epoxy), were fabricated and prepared in accordance with ASTM D3039/D3039M-14, ASTM D3410/D3410M-16, and ASTM D790-17 for tensile, compression, and flexural testing, respectively [57–59].

Three specimens were tested for each laminate configuration under each loading condition in accordance with the adopted ASTM testing procedures, and the reported results represent the arithmetic mean of the measured values. The repeatability of the measured responses was verified through replicate testing, and consistent performance trends were observed among all laminate configurations. No anomalous failure behaviour or significant scatter was observed among the replicate specimens tested under identical loading conditions. All experimental tests were conducted using a computerized Universal Testing Machine (UTM, Jyothi Spectro Testing Equipment Pvt. Ltd., Hyderabad, India). The obtained experimental results were subsequently compared with finite element predictions generated using ANSYS Mechanical APDL 2020 R1 to evaluate the accuracy of the numerical model.

#### Tensile testing.

The primary factors considered while choosing materials for structural purposes are tensile characteristics including strength, modulus & Poisson’s ratio. Tensile testing was performed using a computerized Universal Testing Machine (UTM, 2.5 kN capacity) in accordance with ASTM D3039/D3039M-14, Standard Test Method for Tensile Properties of Polymer Matrix Composite Materials [57]. Fig 2 illustrates the schematic diagram of tensile specimen. The tensile test specimens for J2/C2/J2, J2/G2/J2, and C2/G2/C2 laminates were fabricated in accordance with ASTM D3039/D3039M-14, Standard Test Method for Tensile Properties of Polymer Matrix Composite Materials, as shown in Fig 3. The testing procedure involved securing the specimen between the grips of the testing machine and applying a tensile load until specimen failure occurred. Fractured hybrid laminates after tensile test are shown in Fig 4. The J2/C2/J2 specimen failed at an applied load of 7785 N, with failure occurring near the bottom region of the gauge section. The corresponding failure code is SGM (Long Splitting–Gage–Middle). The J2/G2/J2 specimen failed at an applied load of 3288 N, with failure occurring slightly below the middle of the gauge section. The corresponding failure code was AGM. The C2/G2/C2 specimen failed at an applied load of 11,361 N, with failure occurring near the top region of the specimen. The corresponding failure code was LAT. The corresponding tensile failure codes are summarized in Table 3 [57,60].

**Table 3:** Tensile failure classification codes according to ASTM D3039/D3039M-14 [57].

First Character		Second Character		Third Character	
Failure Type	Code	Failure Area	Code	Failure Location	Code
**J**_**2**_ **/C**_**2**_ **/J**_**2**_					
S	Long Splitting	G	Gage	M	Middle
**J**_**2**_ **/G**_**2**_ **/J**_**2**_					
A	Angled	G	Gage	M	Middle
**C**_**2**_ **/G**_**2**_ **/C**_**2**_					
L	Lateral	A	At grip/tab	T	Top

#### Compression testing.

**Fig 6:**
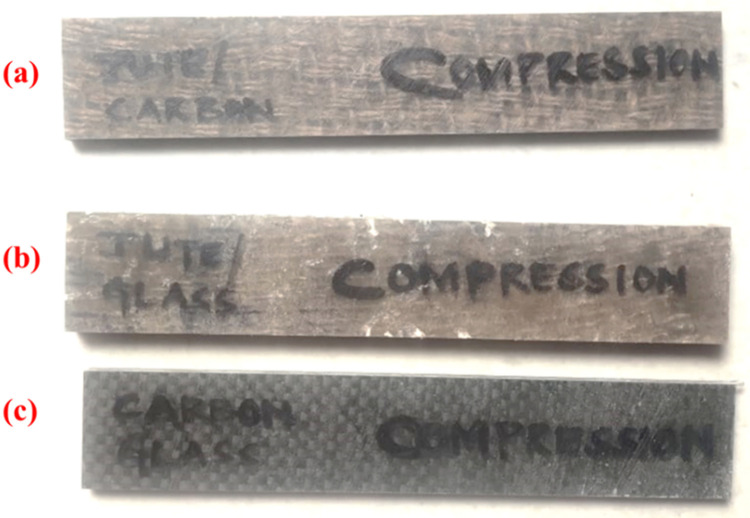
Compression test specimens of (a) J_2_/C_2_/J_2_, (b) J_2_/G_2_/J_2_, and (c) C_2_/G_2_/C_2._

**Fig 7:**
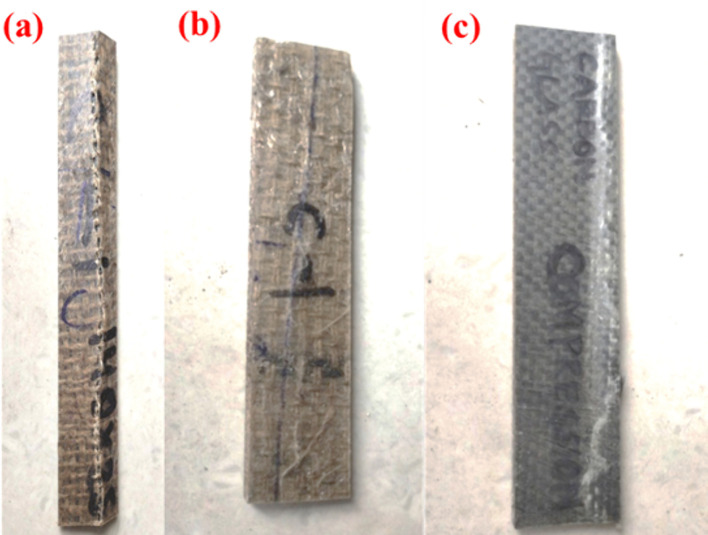
Fractured compression specimens of (a) J2/C2/J2, (b) J2/G2/J2, and (c) C2/G2/C2.

**Fig 8:**
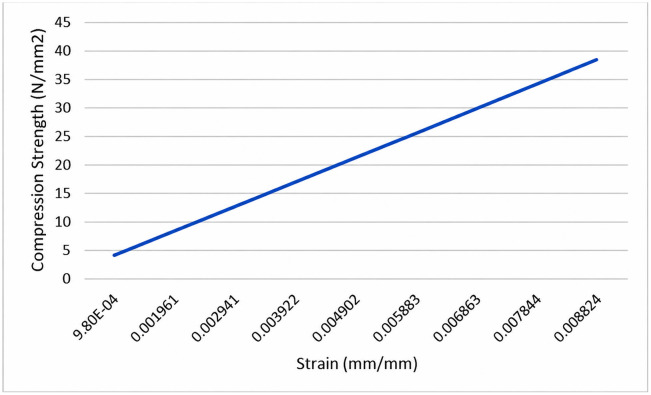
Representative compressive stress–strain response of the J2/C2/J2 laminate obtained from experimental testing.

The matrix provides lateral support and stability to the reinforcing fibers under longitudinal compressive loading and therefore plays an important role in the compressive behavior of composite laminates. Compression testing was carried out using a computerized Universal Testing Machine (UTM, 400 kN capacity) in accordance with ASTM D3410/D3410M-16 [59]. The schematic diagram of the compression test specimen is presented in Fig 5. The compression test specimens for the J2/C2/J2, J2/G2/J2, and C2/G2/C2 laminates were prepared in accordance with ASTM D3410/D3410M-16, Standard Test Method for Compressive Properties of Polymer Matrix Composite Materials by Shear Loading, as shown in Fig 6. The testing procedure involved positioning the specimen within the compression fixture and applying a compressive load until failure occurred. The fractured specimens obtained after compression testing are shown in Fig 7, while the representative compressive stress–strain response is presented in Fig 8.

#### Flexural testing.

**Fig 9:**
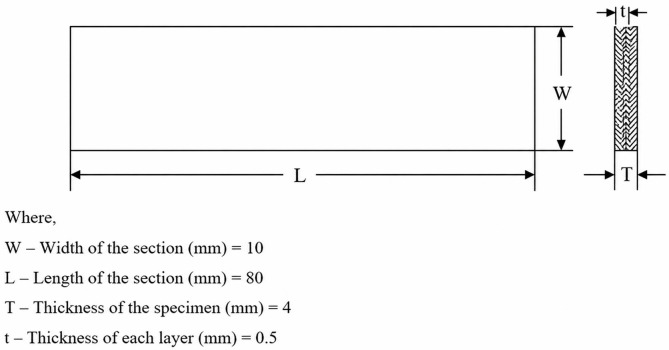
Schematic illustration of flexural specimen according to ASTM D790-17.

**Fig 10:**
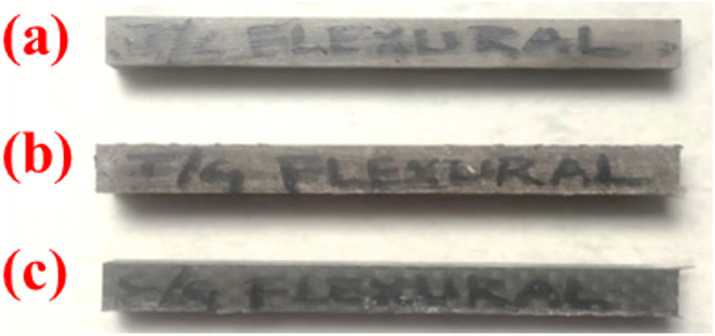
Flexural test specimens of J_2_/C_2_/J_2_, J_2_/G_2_/J_2_, & C_2_/G_2_/C_2._

**Fig 11:**
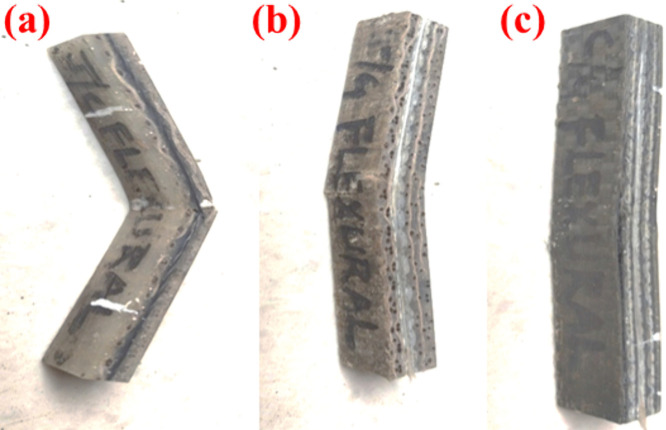
Fractured (a) J_2_/C_2_/J_2_ (b) J_2_/G_2_/J_2_ (c) C_2_/G_2_/C_2_ specimens under flexural test.


Tensile and compressive stresses are developed in the component due to bending under transverse load. The load bearing capacity of the laminate can be found using the flexural test. The most used flexural examination for composite components is the 3-point flexure test. Fig 9 illustrates the schematic diagram of flexural specimen. The flexural test specimens for J2/C2/J2, J2/G2/J2, and C2/G2/C2 laminates were fabricated in accordance with ASTM D790-17 [58], Standard Test Methods for Flexural Properties of Unreinforced and Reinforced Plastics and Electrical Insulating Materials, as shown in Fig 10. The flexural tests were conducted using a Universal Testing Machine (UTM, 600 kN capacity). The testing procedure involved placing the specimen on a three-point bending fixture and applying the load until failure occurred. The test outcomes included the ultimate flexural load and flexural strength of the investigated laminates. Ruptured laminates after flexural testing are shown in Fig 11. The flexural strength (FS) of composite specimen is determined using the Equation 1 [61].

$$\begin{array}{c} \;FS=\frac{\text{3}PL}{{\text{2}bt}^{\text{2}}} \end{array}$$ (1)

Where, P is the load (N) applied. L is the sample span length (mm); b and t are thickness & width of the sample (mm), respectively.

### Numerical approach of HCL

The hybrid composite laminate (HCL) specimens were designed and analyzed in accordance with the relevant ASTM standards using ANSYS Mechanical APDL 2020 R1 (ANSYS Inc., Canonsburg, PA, USA) [62,63]. The numerical analysis was conducted to evaluate the mechanical response of the different laminate configurations and identify the laminate exhibiting the best overall mechanical performance. The orthotropic elastic properties assigned to the laminate plies were adopted from representative literature on jute/epoxy, glass/epoxy, and carbon/epoxy composite systems [47,54,56] and implemented in ANSYS Mechanical APDL for layered composite analysis. Recent reviews on multiscale modelling of natural-fibre-reinforced composites have further highlighted the importance of numerical approaches for understanding the influence of fibre architecture and constituent interactions on composite performance [64]. The selected material constants fall within the ranges commonly reported for woven-fabric composite laminates manufactured using epoxy matrices. The orthotropic elastic properties used in the finite element simulations are summarized in Table 4.

**Table 4:** Orthotropic material properties assigned to the laminate constituents in the finite element model.

Material	E_1_ (GPa)	E_2_ (GPa)	G_12_ (GPa)	ν_12_	Density (kg/m³)
Jute/Epoxy	10.5	3.5	1.8	0.3	1350
Glass/Epoxy	38	8.5	4.2	0.28	1950
Carbon/Epoxy	65	6	4.5	0.3	1600

The orthotropic material properties used in the finite element model were obtained from published literature sources and were not derived, calibrated, or back-calculated from the experimental strength results reported in the present study.

The material properties listed in Table 4 were assigned to the corresponding laminate plies in the finite element model according to the selected stacking sequences. SHELL181, a four-node structural shell element well suited for modeling multilayer composite laminates with orthotropic material behavior and layered shell definitions, was employed for all finite element simulations [62]. Three laminate configurations, namely J2/C2/J2, J2/G2/J2, and C2/G2/C2, each consisting of six plies arranged in an alternating [0°/90°] stacking sequence, were considered. The experimentally measured loads and corresponding boundary conditions were applied to the numerical models to replicate the testing conditions. The finite element predictions were subsequently validated against the experimental results to assess the accuracy and reliability of the numerical approach.

#### Finite element modeling assumptions and boundary conditions.

1. Boundary condition for tensile test using numerical analysis
  (a) One end of the laminate is fixed in all degree of freedom (i.e., in x,y and z directions)
  (b) Load obtained from experimental test are applied on the other end of the laminate.
  (c) Pulling direction of laminate is considered as x-axis
  (d) The element edge length is 3 mm for all the tests.
2. The finite element model was developed to replicate the experimental boundary conditions as closely as possible, and the experimentally measured loads were applied in the numerical simulations for validation purposes.
3. Boundary condition for compression test using numerical analysis
  (a) One end of the laminate is fixed in all degree of freedom (i.e., in x,y and z directions)
  (b) Load obtained from experimental test are applied on the other end of the laminate.
  (c) Pushing direction of laminate is considered as x-axis.
4. Boundary condition for flexural test using numerical analysis
  (a) Three-point load bending condition are assumed.
  (b) Laminate is fixed at y-axis, i.e., 50 mm distance from each end. Load is applied towards the downwards direction from the same axis.

The finite element model assumed perfect interfacial bonding between adjacent laminate layers and did not explicitly account for voids, interfacial debonding, manufacturing defects, or material degradation effects. These simplifications may contribute to minor differences between experimental and numerical results.

#### Grid independence test (GIT).

**Fig 12:**
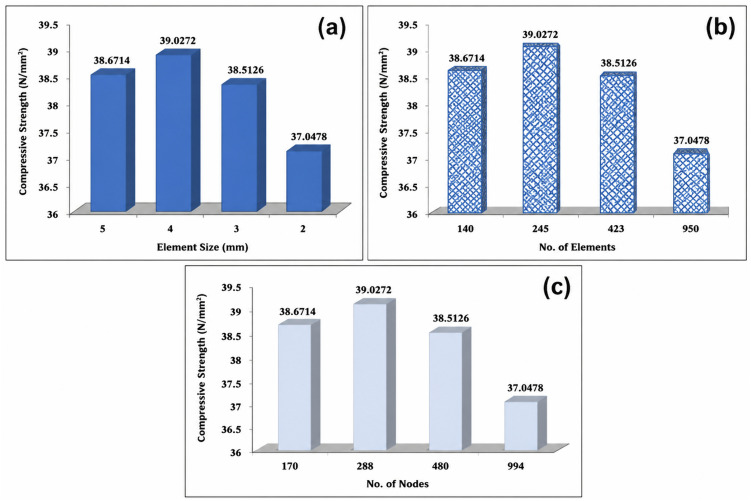
Grid independence assessment showing the variation of predicted von Mises stress with (a) element size, (b) number of elements, and (c) number of nodes.

A grid independence test was conducted to ensure that the numerical results were not significantly influenced by mesh density. The finite element model was initially generated using a coarse mesh and subsequently refined by reducing the element size. For each mesh configuration, the corresponding von Mises stress values were evaluated and compared. Mesh independence was assumed when further mesh refinement resulted in negligible variation in the predicted stress values. The results of the grid independence study are presented in Table 5 and Fig 12. Based on the comparison between numerical and experimental results, an element size of 3 mm was selected for all subsequent finite element analyses as it provided an acceptable balance between computational efficiency and prediction accuracy.

**Table 5:** Grid independence study for the J2/C2/J2 laminate under compression loading.

Element Size (mm)	Number of Elements	Number of Nodes	Predicted von Mises Stress (MPa)
5	140	170	38.671
4	245	288	39.027
3	423	480	38.513
2	910	994	37.048
Experimental compressive strength = 38.571 MPa			

The predicted von Mises stress values exhibited only minor variation with mesh refinement, indicating satisfactory numerical convergence. The variation in predicted stress between the 3 mm and 4 mm mesh sizes was less than 1.5%, confirming mesh convergence. The 3 mm element size produced the closest agreement with the experimentally measured compressive strength (38.571 MPa) and was therefore selected for all subsequent finite element analyses. Consequently, all subsequent numerical simulations were performed using the converged 3 mm mesh configuration.

#### Stress analysis of HCL-Tensile testing.

**Fig 13:**
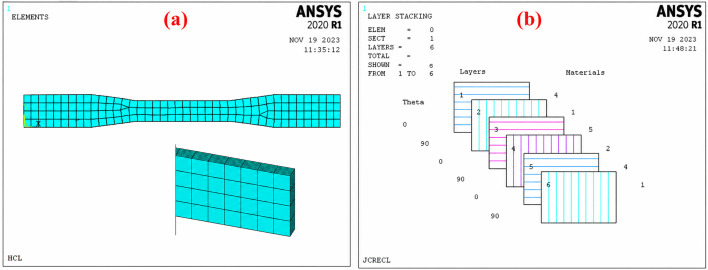
Finite element model used for tensile analysis: (a) meshed tensile specimen and (b) laminate stacking sequence of J2/C2/J2.

**Fig 14:**
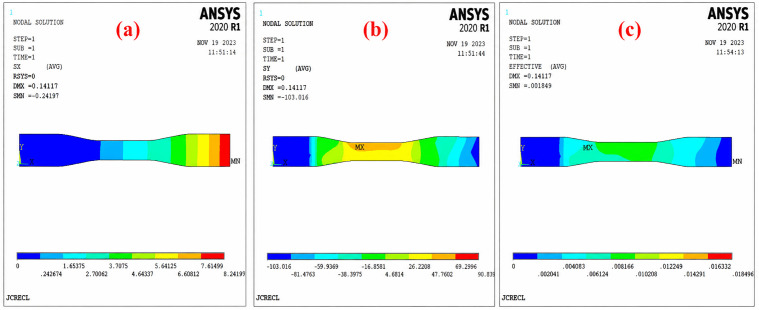
Finite element results for the J2/C2/J2 laminate under tensile loading: (a) total deformation, (b) tensile stress distribution, and (c) tensile strain distribution.

The geometrical meshed model as per ASTM D3039 for tensile testing along with layer stacking of (J_2_/C_2_/J_2_) at a ply orientation of [0°/90°]as shown in Fig 13. Fig 14 shows the placement of the deformation, tensile stress and strain concentrations over the entire specimen. Similarly for the same specimen the analysis is also done with the materials J_2_/G_2_/J_2_ and C_2_/G_2_/C_2_ layer sequence at [0°/90°] ply orientation. The finite element results indicate that the maximum tensile stress concentration occurred within the gauge section of the specimen, which is consistent with the experimentally observed tensile failure locations. The stress and strain distributions further confirm effective load transfer through the laminate under axial tensile loading.

#### Stress analysis of HCL- compression test.

**Fig 15:**
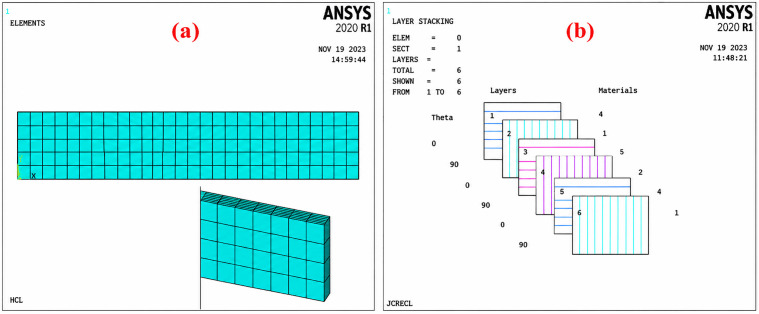
Finite element model used for compression analysis: (a) meshed compression specimen and (b) laminate stacking sequence of J2/C2/J2.

**Fig 16:**
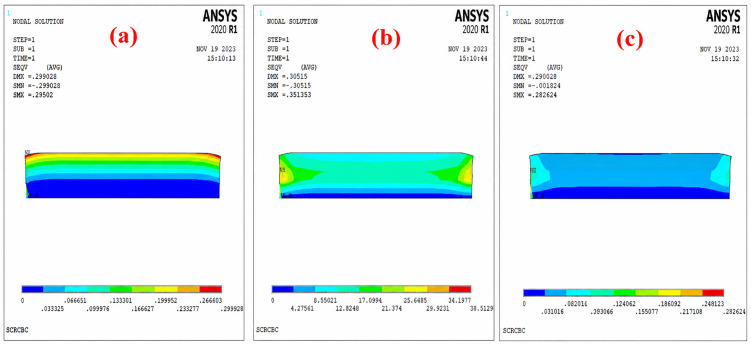
Finite element results for the J2/C2/J2 laminate under compression loading: (a) total deformation, (b) compressive stress distribution, and (c) compressive strain distribution.

The geometrical meshed model as per ASTM D3410 for compression testing along with layer stacking of (J_2_/C_2_/J_2_) at a ply orientation of [0°/90°] is shown in Fig 15. Fig 16 shows the placement of the deformation, compressive stress and strain concentrations over the entire specimen. Similarly for the same specimen the analysis is also done with the materials J_2_/G_2_/J_2_ and C_2_/G_2_/C_2_ at [0°/90°] angle orientations. The compressive stress contours indicate a relatively uniform stress distribution within the central region of the laminate, while localized stress concentrations developed near the constrained regions. The numerical results demonstrate stable load transfer through the laminate thickness under compressive loading conditions.

#### Stress analysis of HCL – Flexural test.

**Fig 17:**
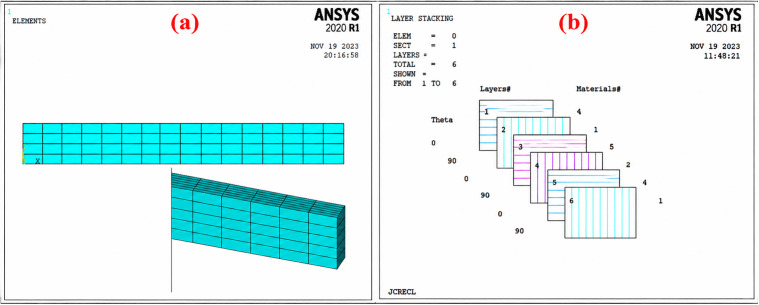
Finite element model used for flexural analysis: (a) meshed flexural specimen and (b) laminate stacking sequence of J2/C2/J2.

**Fig 18:**
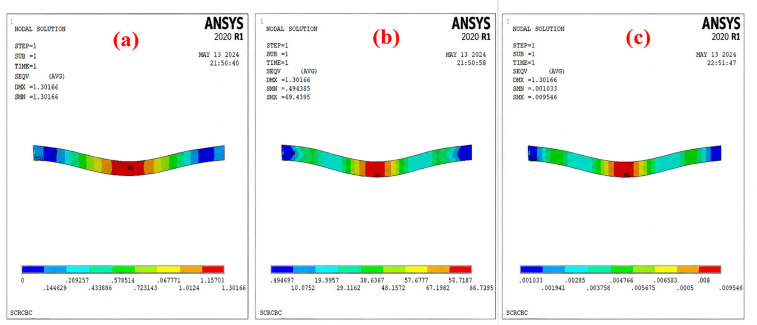
Finite element results for the J2/C2/J2 laminate under flexural loading: (a) total deformation, (b) flexural stress distribution, and (c) flexural strain distribution.

The finite element model developed according to ASTM D790 for flexural testing, together with the laminate stacking sequence of J2/C2/J2 at a [0°/90°] ply orientation, is presented in Fig 17. Fig 18 illustrates the distributions of total deformation, flexural stress, and flexural strain throughout the specimen under bending loading. Similar analyses were performed for the J2/G2/J2 and C2/G2/C2 laminate configurations using the same [0°/90°] ply orientation and boundary conditions. Under flexural loading, the highest stress concentrations were observed near the outer surfaces of the laminate, where maximum tensile and compressive bending stresses are expected. The deformation pattern obtained from the numerical model is consistent with the behavior of a simply supported laminate subjected to three-point bending.

## Results and discussion

The mechanical response of hybrid composite laminates is strongly influenced by fiber type, stacking sequence, and ply orientation. The interaction between the reinforcing fibers and the matrix governs the load-transfer mechanisms and ultimately determines the tensile, compressive, and flexural behavior of the laminate systems investigated in the present study.

### Experimental results

**Fig 19:**
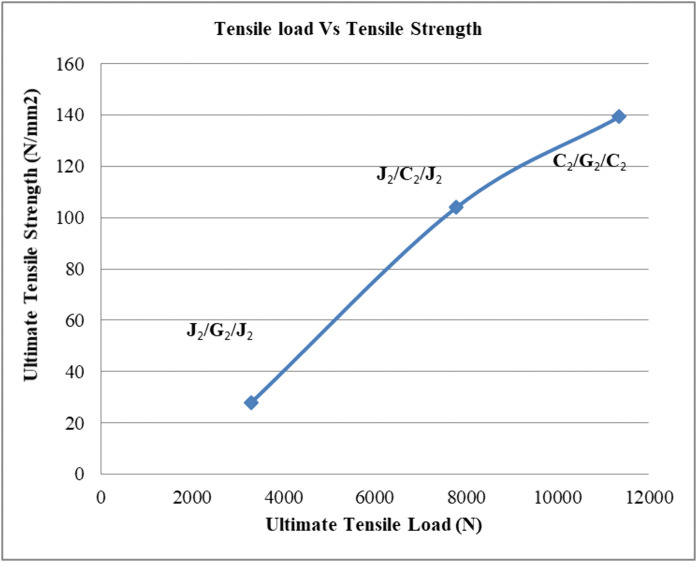
Comparison of ultimate tensile load and tensile strength for the investigated hybrid laminate configurations.

The experimental outcomes are clearly represented in Fig 19. From the results it is clearly shown that C_2_/G_2_/C_2_ laminate performs better in terms of tensile strength than the other hybrid laminates, as evidenced by its highest ultimate tensile load (U.T.L) & ultimate tensile strength (U.T.S). J_2_/G_2_/J_2_ has somewhat lower values, although J_2_/C_2_/J_2_ likewise has significant U.T.L and U.T.S values. Glass fibers are usually less expensive & have superior strength compared to carbon fibers, which are known for their great strength and stiffness. These fibers combined might provide a well-balanced blend of high strength and stiffness in the laminate. The fibers’ precise orientation and stacking order play a part in how well the laminate withstands the loads. By uniting the special qualities of both the fiber types, the glass & carbon fibers in C_2_/G_2_/C_2_ probably have a synergistic effect.

High U.T.L. and U.T.S. values are also shown by (J_2_/C_2_/J_2_), indicating robust tensile performance. The laminate’s total strength is affected by the order and combination of carbon and jute fibers. Carbon fibers deliver high strength and stiffness, jute fibers, being natural, contribute to a sustainable environment and may boost impact resistance. A composite material that combines natural and synthetic fibers can reap the benefits of each material’s distinct qualities, producing an overall performance that is well-balanced.

Comparatively speaking, (J_2_/G_2_/J_2_) has lower U.T.L and U.T.S values. Glass fibers give further strength and rigidity, while jute fibers contribute to sustainability in the natural world. The goal of the combination is to maximize the advantages of both kinds of fiber. The present observations are consistent with previous investigations on hybrid composite laminates reported in the literature. Dong and Davies [46] demonstrated that carbon–glass hybridization can significantly improve stiffness and load-carrying capability compared with single-fiber composite systems. Similarly, Zhang et al. [49] reported that hybrid laminates incorporating carbon-fiber outer plies exhibited superior mechanical performance owing to the higher stiffness and strength of carbon reinforcement. The improved tensile behavior observed for the C2/G2/C2 laminate in the present study therefore agrees well with previously reported trends for carbon–glass hybrid laminate architectures.

The observed tensile behavior highlights the importance of laminate architecture in addition to the intrinsic properties of the reinforcing fibers. In the C2/G2/C2 configuration, the carbon-fiber outer plies are positioned in the regions subjected to the highest tensile stresses, enabling efficient load transfer and improved stiffness. In contrast, the incorporation of jute-fiber outer layers in J2/C2/J2 and J2/G2/J2 reduces the overall tensile resistance because the lower stiffness of natural fibers limits the load-carrying capability of the laminate. These findings indicate that both fiber type and stacking arrangement play important roles in determining tensile performance. Furthermore, the glass-fiber core contributes to improved stress redistribution between the outer carbon-fiber layers, reducing local stress concentrations and promoting more uniform load transfer across the laminate thickness. This synergistic interaction between carbon and glass fibers is considered a major factor responsible for the superior overall mechanical performance of the C2/G2/C2 configuration

**Fig 20:**
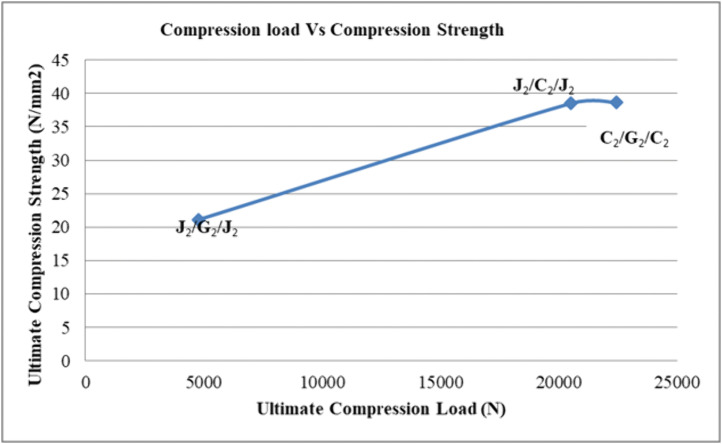
Comparison of ultimate compressive load and compressive strength for the investigated hybrid laminate configurations.

The experimental compression load versus strength results are presented in Fig 20. Among the investigated laminate configurations, C2/G2/C2 exhibited the highest compressive strength, whereas J2/G2/J2 showed the lowest performance. The observed differences indicate that compressive behavior is influenced not only by the constituent materials but also by laminate architecture and stacking sequence. Under compressive loading, the performance of hybrid laminates depends on their ability to resist deformation while maintaining structural stability. The superior response of the C2/G2/C2 laminate can be attributed to the higher stiffness of carbon-fiber plies, which contributes to improved load-bearing capability under compression. In contrast, the comparatively lower performance of the jute-containing laminates may be associated with the lower stiffness of natural fibers, resulting in reduced resistance to compressive deformation. The comparatively lower compressive strengths relative to the corresponding tensile strengths are consistent with the typical behavior of fiber-reinforced polymer laminates. Unlike tensile loading, where fibers carry the majority of the applied load, compressive loading is often governed by matrix-dominated failure mechanisms. Localized fiber microbuckling, matrix yielding, interfacial instability, and premature delamination can develop before the full load-carrying capacity of the fibers is reached. Consequently, compressive failure generally occurs at lower stress levels than tensile failure. The superior compressive performance of the C2/G2/C2 laminate may therefore be attributed to the higher stiffness of the carbon-fiber outer plies, which improved resistance to compressive deformation and enhanced overall structural stability.

**Fig 21:**
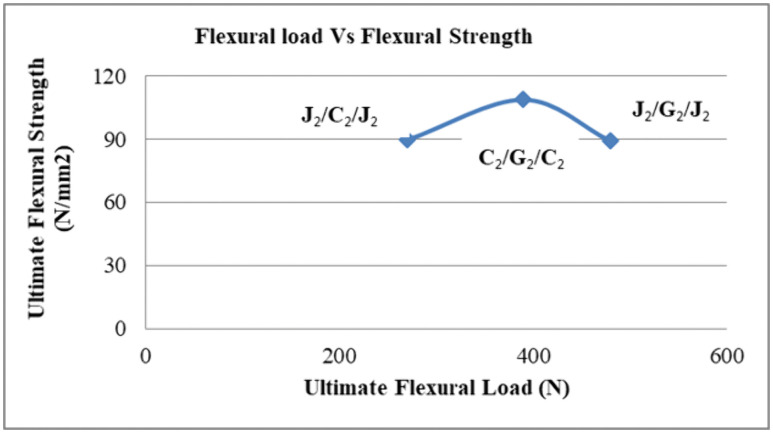
Comparison of ultimate flexural load and flexural strength for the investigated hybrid laminate configurations.

The experimental flexural test results are presented in Fig 21. Among the investigated laminate configurations, C2/G2/C2 exhibited the highest flexural strength, followed by J2/C2/J2 and J2/G2/J2. The flexural response of hybrid laminates is governed by the combined tensile and compressive stresses generated across the laminate thickness during bending. Since the outer plies experience the highest stresses, their mechanical properties strongly influence the overall flexural behavior. The superior flexural performance of the C2/G2/C2 laminate indicates that the placement of carbon-fiber plies on the outer surfaces enhances bending resistance and stiffness. Furthermore, the results demonstrate that laminate stacking sequence and fiber arrangement play significant roles in determining flexural performance. These findings highlight the importance of hybrid laminate architecture in improving structural behavior under bending loads. Similar findings have been reported in earlier studies on hybrid composite laminates. Zhang et al. [49] and Dong [52] observed that positioning carbon-fiber layers near the outer surfaces of a laminate significantly enhances flexural performance because the outer regions experience the highest tensile and compressive stresses during bending. The superior flexural response of the C2/G2/C2 laminate obtained in the present investigation is therefore consistent with the established behavior of carbon–glass hybrid laminate systems.

### Numerical & experimental results comparison

**Fig 22:**
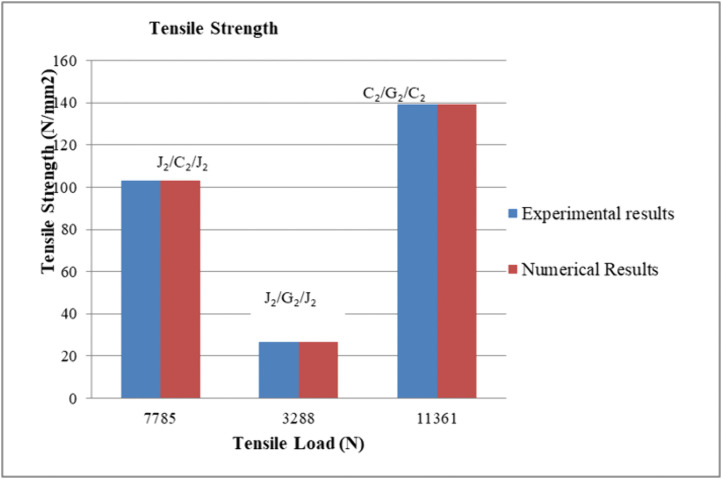
Comparison of experimental and numerical tensile strength results.

**Fig 23:**
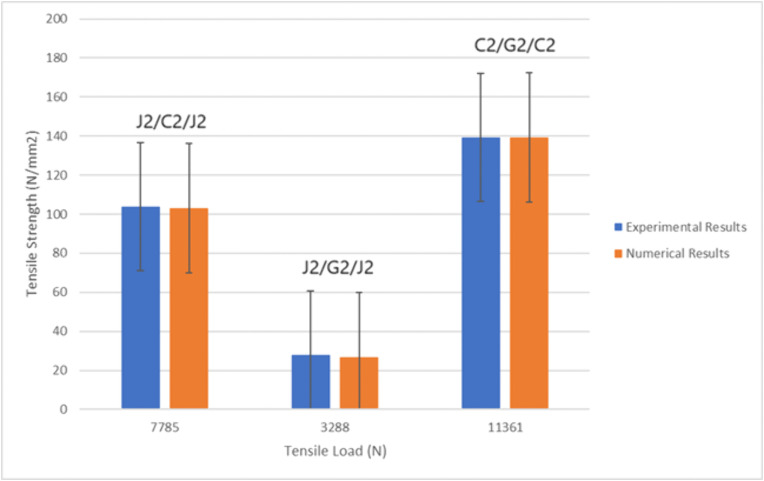
Percentage error between experimental and numerical tensile strength results.

**Fig 24:**
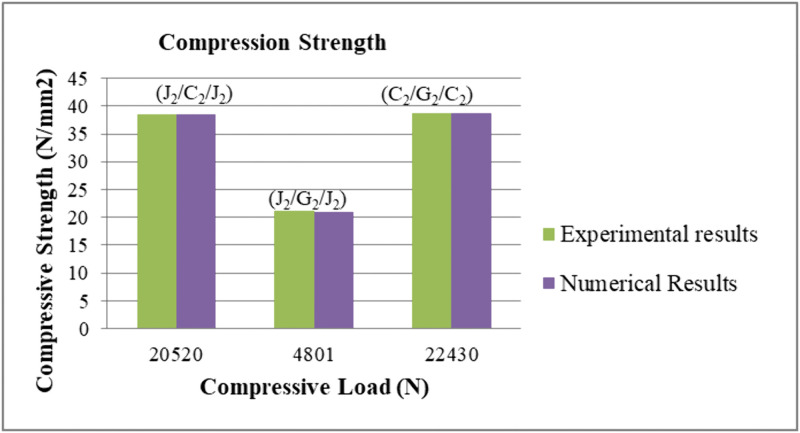
Comparison of experimental and numerical compressive strength results.

**Fig 25:**
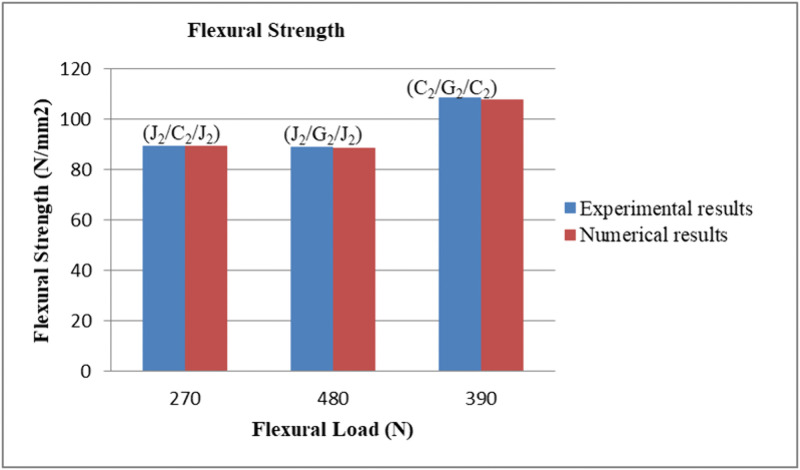
Comparison of experimental and numerical flexural strength results.

The finite element predictions demonstrated good agreement with the experimentally measured mechanical properties for all laminate configurations investigated in the present study. It is observed that both the analysis undergone minimal differences as shown in bar diagrams in Figs 22-25.

The bar diagram below in Figs 22, 23 shows difference between the experimental and numerical analysis for tensile strength and error graph, where we can observe the percentage error of J_2_/C_2_/J_2_ having 0.86%, J_2_/G_2_/J_2_ of 1.13% and C_2_/G_2_/C_2_ of 0.04% these values are also tabulated in Table 6. The J2/C2/J2 and C2/G2/C2 laminates exhibited superior mechanical performance compared with the J2/G2/J2 laminates. The strength difference between the C_2_/G_2_/C_2_ and J_2_/C_2_/J_2_ in percentage is 29.116%.

**Table 6:** Error percentage of Tensile strength in comparison between Experimentation and Numerical analysis.

Type of HCL	Error percentage of Tensile strength in comparison between Experimentation and Numerical analysis
	Error (%)
J_2_/C_2_/J_2_	0.86
J_2_/G_2_/J_2_	1.13
C_2_/G_2_/C_2_	0.04

As shown in Table 6, the percentage error between experimental and numerical tensile strength remained below 1.2% for all laminate configurations.

The bar diagram below in Fig 24 shows difference between the experimental and numerical analysis for compressive strength where we can observe the percentage error of (J_2_/C_2_/J_2_) having 0.15%, (J_2_/G_2_/J_2_) of 0.46% and (C_2_/G_2_/C_2_) of 0.12% these values are also tabulated in Table 7. In terms of compressive strength, the combination of (J_2_/C_2_/J_2_) and (C_2_/G_2_/C_2_) have shown good results compared to (J_2_/G_2_/J_2_). The strength difference between the (C_2_/G_2_/C_2_) and (J_2_/C_2_/J_2_) in percentage is 0.33%.

**Table 7:** Error percentage of compression strength in comparison between experimentation and numerical analysis.

Type of HCL	Error percentage of Compression strength in comparison between Experimentation and Numerical analysis
	Error (%)
(J_2_/C_2_/J_2_)	0.15
(J_2_/G_2_/J_2_)	0.46
(C_2_/G_2_/C_2_)	0.12

Table 7 further confirms the close agreement between experimental and numerical compressive strength predictions.

The bar diagram below in Fig 25, shows difference between the experimental and numerical analysis for flexural strength where we can observe the percentage error of (J_2_/C_2_/J_2_) having 0.19%, (J_2_/G_2_/J_2_) of 0.52% and (C_2_/G_2_/C_2_) of 0.57% these values are also tabulated in Table 8. In terms of flexural strength, the combination of (J_2_/C_2_/J_2_) and (C_2_/G_2_/C_2_) have shown good results compared to (J_2_/G_2_/J_2_). The strength difference between the (C_2_/G_2_/C_2_) and (J_2_/C_2_/J_2_) in percentage is 19.29%.

**Table 8:** Error percentage of Flexural strength in comparison between experimentation and numerical analysis.

Type of HCL	Error percentage of Flexural strength in comparison between Experimentation and Numerical analysis
	Error (%)
(J_2_/C_2_/J_2_)	0.19
(J_2_/G_2_/J_2_)	0.52
(C_2_/G_2_/C_2_)	0.57

The low error values reported in Table 8 demonstrate the capability of the finite element model to accurately predict flexural strength. Fig 25 demonstrates that the finite element predictions closely follow the experimentally measured flexural strengths for all laminate configurations, with only minor deviations between the numerical and experimental results. The close agreement between experimental and numerical results demonstrates that the finite element model was capable of capturing the overall mechanical response of the investigated laminates with satisfactory accuracy. The maximum deviation remained below 1.2% under the adopted modelling assumptions, indicating that the selected material properties, boundary conditions, and mesh configuration were adequate for comparative performance evaluation. The relatively small deviations may be attributed to the simplified specimen geometries, controlled loading conditions, and the application of experimentally measured loads in the numerical simulations. Nevertheless, the reported agreement should not be interpreted as universal predictive accuracy because additional discrepancies may arise under more complex loading conditions, larger structural components, or when manufacturing-induced defects and progressive damage mechanisms are explicitly considered. It should be noted that the finite element model assumed perfect interfacial bonding between adjacent plies and did not explicitly account for voids, manufacturing defects, progressive damage evolution, or interfacial debonding. Therefore, the numerical predictions should be interpreted as comparative estimates of laminate behavior rather than exact representations of physical failure mechanisms. The results further confirm that numerical analysis can be effectively used as a preliminary design tool for assessing hybrid laminate configurations prior to fabrication and testing. The close agreement between experimental and numerical results should be interpreted within the limitations of the adopted modelling framework. The finite element model utilized literature-derived orthotropic elastic properties together with experimentally applied loading conditions and assumed perfect interfacial bonding between adjacent laminate layers. Manufacturing defects, voids, local material heterogeneity, and progressive damage evolution were not explicitly considered. Therefore, the numerical model is intended primarily for comparative performance assessment rather than detailed failure prediction.

The results indicate that the incorporation of carbon fiber into jute-based laminates produced an average strength improvement of approximately 88%, whereas glass-fiber incorporation resulted in an average improvement of approximately 55% compared with the jute-based laminate configuration [65]. The observed differences among the C2/G2/C2, J2/C2/J2, and J2/G2/J2 laminates demonstrate the beneficial influence of hybridization on mechanical performance. Among the investigated configurations, C2/G2/C2 consistently exhibited the highest tensile, compressive, and flexural strengths, indicating superior overall structural performance. This behavior may be attributed to the high stiffness and load-carrying capability of carbon-fiber reinforcement combined with the complementary contribution of the glass-fiber layers.

The observed correlation can be attributed to the simplified laminate geometry, controlled loading conditions, and the application of experimentally measured loads in the numerical simulations. Although minor deviations are unavoidable because of material variability, manufacturing imperfections, and modeling assumptions, the overall agreement confirms the suitability of the adopted finite element approach for comparative assessment of hybrid laminate configurations.

The reported mechanical properties represent the arithmetic mean of three specimens tested for each laminate configuration. The experimental results exhibited consistent performance trends among all laminate systems, and no anomalous failure behavior was observed during testing. Although the number of specimens was limited, the repeatability of the measured responses provided confidence in the observed ranking of laminate performance. Nevertheless, additional testing involving a larger sample size and comprehensive statistical analyses would further strengthen the statistical confidence and provide deeper insight into result variability and repeatability.

The present study was limited to the quasi-static tensile, compressive, and flexural characterization of flat hybrid composite laminates together with finite element validation. Void content was not experimentally quantified, and microscopic failure characterization such as scanning electron microscopy (SEM) was not performed. Consequently, detailed failure mechanisms including fiber breakage, matrix cracking, interfacial debonding, and delamination could not be directly examined. Failure assessment was therefore based on macroscopic examination of the fractured specimens obtained after tensile, compressive, and flexural testing. Although these observations provided qualitative evidence of laminate failure behaviour, detailed microstructural failure mechanisms could not be conclusively identified without SEM characterization.

The experimental program was restricted to three specimens for each laminate configuration under each loading condition. Although consistent performance trends were observed among all laminate systems, future investigations involving larger sample sizes and comprehensive statistical analyses would further improve the reliability and statistical confidence of the reported results.

The finite element model assumed linear elastic orthotropic material behavior, perfect interfacial bonding between adjacent plies, and the absence of manufacturing defects or voids. Progressive damage evolution, interlaminar failure, delamination growth, and advanced composite failure criteria were not considered. Therefore, the numerical model should be interpreted primarily as a comparative engineering tool rather than a comprehensive failure-prediction framework.

In addition, interlaminar shear strength, fatigue performance, impact resistance, environmental aging, moisture absorption, creep behavior, and full-scale pressure-vessel testing were beyond the scope of the present investigation. These aspects should be addressed in future studies to establish the long-term structural reliability and practical applicability of the investigated hybrid laminate systems.

Additional numerical data, finite element input parameters, and validation results are provided in Supporting Information (S1 File).

The findings indicate that the investigated laminates possess considerable potential for lightweight engineering applications requiring enhanced mechanical performance. In particular, the superior behavior of the C2/G2/C2 configuration suggests that it may be a promising candidate for future investigation in lightweight composite structures. However, its suitability for pressure-vessel applications requires dedicated pressure-vessel fabrication and internal-pressure testing.

## Conclusions

The present investigation combined experimental characterization and finite element analysis to evaluate the mechanical performance of three natural–synthetic hybrid composite laminate configurations, namely J2/C2/J2, J2/G2/J2, and C2/G2/C2. The results demonstrate that hybridization provides an effective approach for improving the mechanical behavior of composite laminates through the strategic combination of natural and synthetic fiber reinforcements. Based on the obtained results, the following conclusions can be drawn:

a) Among the investigated laminate configurations, C2/G2/C2 exhibited the highest tensile, compressive, and flexural strengths, indicating its superior overall mechanical performance
b) Although C2/G2/C2 demonstrated the best mechanical performance, J2/C2/J2 exhibited competitive properties while incorporating a natural-fiber component, making it an attractive alternative where sustainability considerations are important.
c) Although J2/C2/J2 and J2/G2/J2 exhibited lower strengths than C2/G2/C2, their mechanical performance may still be adequate for applications where sustainability, reduced material cost, or balanced structural properties are important design considerations.
d) The results demonstrate that appropriate hybridization of jute, glass, and carbon fibers can significantly improve the mechanical performance of composite laminates compared with natural-fiber-dominated laminate systems.
e) Numerical predictions showed good agreement with experimental measurements, with maximum deviations below 1.2% under the adopted modelling assumptions, supporting the suitability of the adopted finite element methodology for comparative assessment of hybrid laminate configurations.
f) The superior performance of the C2/G2/C2 laminate highlights the beneficial role of carbon-fiber outer plies in enhancing the tensile, compressive, and flexural response of hybrid composite laminates. The results demonstrate that both fiber selection and laminate architecture significantly influence the overall mechanical performance of hybrid composite systems.
g) The present investigation was limited to flat laminate coupon testing and finite element validation. Therefore, the suitability of the investigated laminates for pressure-vessel applications requires dedicated pressure-vessel fabrication, burst-pressure evaluation, and long-term durability assessment.

Based on the observed mechanical performance, the C2/G2/C2 and J2/C2/J2 laminate configurations demonstrated promising characteristics for lightweight composite components requiring enhanced strength and stiffness. The investigated laminates with a [0°/90°] ply orientation exhibited satisfactory tensile, compressive, and flexural performance under the applied loading conditions. Future investigations should focus on fatigue behavior, impact resistance, environmental durability, moisture absorption, and full-scale composite pressure-vessel testing to establish the long-term structural reliability of these hybrid composite systems. These findings indicate that the investigated laminates possess promising characteristics for lightweight structural composite applications requiring enhanced mechanical performance. Among the investigated configurations, the C2/G2/C2 laminate exhibited the most favorable overall tensile, compressive, and flexural performance and therefore represents the most promising candidate for future lightweight composite structural applications. The suitability of these laminates for pressure-vessel applications cannot be established based on the present investigation alone and requires dedicated pressure-vessel fabrication, internal-pressure testing, burst-pressure evaluation, and long-term durability assessment.

## Supporting information

S1 File Finite element modelling input parameters, additional numerical results, and validation data used in the present study. (DOCX)

## References

[1] Sadaq SI, Kumar VS, Ahmed GMS, Irfan Md. Experimental Investigation and Impact Analysis of GFRP Composite Laminates. Materials Today: Proceedings. 2015;2(4–5):2808–16. doi: 10.1016/j.matpr.2015.07.291

[2] Khawale VR, Musrif PG, Agarwal A, Seenivasan M, Saminathan R, Satishkumar P, et al. Experimental and numerical investigation of low-velocity impact response of piassava fiber composite sandwich panels. Interactions. 2026;247(1). doi: 10.1007/s10751-026-02517-7

[3] Fang H, Xi L, Huang Y, Zhao T, He R, Kang X, et al. Fiber-reinforced composites: A comprehensive review of traditional and additive manufacturing processes and material architectures. Composites Part A: Applied Science and Manufacturing. 2026;205:109684. doi: 10.1016/j.compositesa.2026.109684

[4] Mulenga TK, Rangappa SM, Lai CW, Anam K, Moure MM, Siengchin S. Synergistic performance in natural fiber hybrid composites: A review of weathering, thermal, and mechanical properties through filler integration. Journal of Materials Research and Technology. 2026;40:662–78. doi: 10.1016/j.jmrt.2025.12.077

[5] Mohammed M, Oleiwi JK, Mohammed AM, Jawad AJM, Osman AF, Adam T, et al. A Review on the Advancement of Renewable Natural Fiber Hybrid Composites: Prospects, Challenges, and Industrial Applications. JRM. 2024;12(7):1237–90. doi: 10.32604/jrm.2024.051201

[6] Safri SNA, Sultan MTH, Jawaid M, Jayakrishna K. Impact behaviour of hybrid composites for structural applications: A review. Compos B Eng. 2018;133:112–21. doi: 10.1016/j.compositesb.2017.09.008

[7] Rajak D, Pagar D, Menezes P, Linul E. Fiber-Reinforced Polymer Composites: Manufacturing, Properties, and Applications. Polymers. 2019;11:1667. doi: 10.3390/polym11101667 31614875 PMC6835861

[8] Venkateshwar Reddy C, Ramesh Babu P, Ramnarayanan R, Das D. Mechanical Characterization Of Unidirectional Carbon And Glass/Epoxy Reinforced Composites For High Strength Applications. Materials Today: Proceedings. 2017;4(2):3166–72. doi: 10.1016/j.matpr.2017.02.201

[9] Letsatsi MT, Agarwal A. Study the effects of dimensional parameter using free vibrational modal analysis of composite laminate. 2022. 899–907. doi: 10.1007/978-981-19-0244-4_83

[10] Patro B, Shashidhar D, Rajeshwer B, Padhi SK. Preparation and Testing of PAN Carbon/Epoxy Resin Composites. TOMEJ. 2017;11(1):14–24. doi: 10.2174/1874155x01711010014

[11] Bianchi T, Naciri J, Serra J, Bouvet C, Ratsifandriahana L. Evaluation of the strain gradient effect on compressive failure of CRFP composites. Composites Part C: Open Access. 2025;17:100621. doi: 10.1016/j.jcomc.2025.100621

[12] Khan ZI, Arsad A, Mohamad Z, Habib U, Zaini MAA. Comparative study on the enhancement of thermo-mechanical properties of carbon fiber and glass fiber reinforced epoxy composites. Materials Today: Proceedings. 2021;39:956–8. doi: 10.1016/j.matpr.2020.04.223

[13] Rahmani H, Najafi SHM, Saffarzadeh‐Matin S, Ashori A. Mechanical properties of carbon fiber/epoxy composites: Effects of number of plies, fiber contents, and angle‐ply layers. Polym Eng Sci. 2014;54:2676–82. doi: 10.1002/pen.23820

[14] Rahmani H, Najafi SHM, Ashori A. Mechanical performance of epoxy/carbon fiber laminated composites. Journal of Reinforced Plastics and Composites. 2014;33(8):733–40. doi: 10.1177/0731684413518255

[15] Ciprian L, Radu P, Ioana E. The Effects of Fibre Volume Fraction on a Glass-Epoxy Composite Material. INCAS BULLETIN. 2015;7(3):113–9. doi: 10.13111/2066-8201.2015.7.3.10

[16] Truong TC, Vettori M, Lomov S, Verpoest I. Carbon composites based on multi-axial multi-ply stitched preforms. Part 4. Mechanical properties of composites and damage observation. Composites Part A: Applied Science and Manufacturing. 2005;36(9):1207–21. doi: 10.1016/j.compositesa.2005.02.004

[17] Bezzou A, Péron M, Casari P, Singery V, Ponsolle D, Jacquemin F. Characterization of microcracking of NCF composites under accelerated hygrothermal cycles: Influence of the stitching yarn and the style of biaxial NCF. Composites Part A: Applied Science and Manufacturing. 2021;149:106507. doi: 10.1016/j.compositesa.2021.106507

[18] Hopped TSJD. Effect of an angle-ply orientation on compression strength of composite laminates. Army Research Laboratory. In: 1999. https://apps.dtic.mil/sti/tr/pdf/ADA368034.pdf

[19] Irfan Sadaq S, Romana S, Lakshmi Kumari NBV, Prasanna Kumar G, Shahar Banu S. Analysis of optimum stacking sequence of GFRP composite laminate under axial loading condition. Materials Today: Proceedings. 2022;62:2940–5. doi: 10.1016/j.matpr.2022.02.510

[20] Irfan Sadaq S, Khadar Vali S, Imran Sharif S. Investigation of hybridized composite pressure vessel. E3S Web Conf. 2021;309:01157. doi: 10.1051/e3sconf/202130901157

[21] Srividya DV, Shaik KV, Hadya B, Sadaq SI. Failure analysis of GFRP and CFRP composite laminated pressure vessel. 2024;:100002. doi: 10.1063/5.0193899

[22] Srividya DV, Khadar Vali S, Hadya B, Irfan Sadaq S. Failure analysis of composite pressure vessel by means of natural and synthetic fibers. Materials Today: Proceedings. 2023. doi: 10.1016/j.matpr.2023.07.287

[23] Ogunleye RO, Rusnakova S, Zaludek M, Emebu S. The Influence of Ply Stacking Sequence on Mechanical Properties of Carbon/Epoxy Composite Laminates. Polymers (Basel). 2022;14(24):5566. doi: 10.3390/polym14245566 36559933 PMC9786175

[24] Zhang Z, Hou S, Mao Y, He L, Han X. Rate-related study on the ply orientation of carbon fiber reinforced epoxy composite laminates. Int J Mech Sci. 2020;188:105968. doi: 10.1016/j.ijmecsci.2020.105968

[25] Baharvand A, Teuwen JJE, Shankar Verma A. A Review of Damage Tolerance and Mechanical Behavior of Interlayer Hybrid Fiber Composites for Wind Turbine Blades. Materials (Basel). 2025;18(10):2214. doi: 10.3390/ma18102214 40428951 PMC12112813

[26] Basha M, Wagih A, Melaibari A, Lubineau G, Eltaher MA. On the impact damage resistance and tolerance improvement of hybrid CFRP/Kevlar sandwich composites. Microporous and Mesoporous Materials. 2022;333:111732. doi: 10.1016/j.micromeso.2022.111732

[27] Iqbal RM, Ahammad R, Arifuzzaman M, Islam MS, Islam MM. Manufacturing and Properties of Jute Fiber-Reinforced Polymer Composites-A Comprehensive Review. Materials (Basel). 2025;18(5):1016. doi: 10.3390/ma18051016 40077241 PMC11901065

[28] Cionita T, Hamdan MHM, Siregar JP, Fitriyana DF, Junid R, Shing WL, et al. Overview of Jute Fibre as Thermoplastic Matrix Polymer Reinforcement. JRM. 2024;12(3):457–83. doi: 10.32604/jrm.2024.045814

[29] Anto T, Rajendran RC, Agarwal A, Jayamani E, Natarajan VD. Investigation of Mechanical Properties of 3D Printed Biodegradable Polylactic Acid Reinforced with Paper Microcrystalline Cellulose. Applied Science and Engineering Progress. 2024. doi: 10.14416/j.asep.2024.08.007

[30] Sharma H, Arora G, Singh MK, Rangappa SM, Bhowmik P, Kumar R, et al. From composition to performance: Structural insights into polymer composites. Next Materials. 2025;8:100852. doi: 10.1016/j.nxmate.2025.100852

[31] Arora G, Sharma H, Bhowmik P, Kumar M, Ayyappan V, Singh MK. From plant fiber to product: Fabrication, properties, and circular pathways of bio-based composites. J Compos Mater. 2026. doi: 10.1177/00219983261425772

[32] Ayyappan V, Arora G, Kumar M, Raghunathan V, Rangappa SM, Siengchin S. Sustainable Composite Products: Industry 4.0 to 5.0. Applied Science and Engineering Progress. 2025. doi: 10.14416/j.asep.2025.07.001

[33] Techawinyutham L, Ayyappan V, Kumar M, Raghunathan V, Rangappa SM, Siengchin S. Sustainable epoxy composites from hemp/pineapple/glass fibers for lightweight automobile panels. Int J Biol Macromol. 2026;337(Pt 1):149392. doi: 10.1016/j.ijbiomac.2025.149392 41344458

[34] Raghavendra G, Ojha S, Acharya S, Pal S. Jute fiber reinforced epoxy composites and comparison with the glass and neat epoxy composites. J Compos Mater. 2014;48:2537–47. doi: 10.1177/0021998313499955

[35] Ganeshkumar S, Felix Sahayaraj A, Jaganathan M, Ramesh M. Coated fiber–reinforced polymer composites for construction applications. Surface Modification and Coating of Fibers, Polymers, and Composites. Elsevier. 2025. 521–41. doi: 10.1016/b978-0-443-22029-6.00025-3

[36] Kaufmann J, Temesgen AG, Cebulla H. A comprehensive review on natural fiber reinforced hybrid composites processing techniques, material properties and emerging applications. Discov Mater. 2025;5:227. doi: 10.1007/s43939-025-00419-z

[37] Mahmud SH, Akram MdW, Ferdous SMdR, Islam D, Fatema K, Chowdhury MdSA, et al. Fabrication and mechanical performance investigation of jute/glass fiber hybridized polymer composites: Effect of stacking sequences. Next Materials. 2024;5:100236. doi: 10.1016/j.nxmate.2024.100236

[38] Mohinoddin M, Irfan Sadaq S, Romana S, Suvarna Kumar V. Experimental characterization of unidirectional carbon – Carbon composite laminate. Materials Today: Proceedings. 2023. doi: 10.1016/j.matpr.2023.07.079

[39] Banakar P. Preparation and characterization of the carbon fiber reinforced epoxy resin composites. IOSR Journal of Mechanical and Civil Engineering. 2012;1:15–8. doi: 10.9790/1684-0131518

[40] Fouda H, Guo L, Elsharkawy K. Preparation and Characterizations of Composite Material Based on Carbon Fiber and Two Thermoset Resins. MATEC Web Conf. 2016;88:02002. doi: 10.1051/matecconf/20178802002

[41] Muralidhara B, Kumaresh Babu SP, Suresha B. The effect of fiber architecture on the mechanical properties of carbon/epoxy composites. Materials Today: Proceedings. 2020;22:1755–64. doi: 10.1016/j.matpr.2020.03.008

[42] Burley A, Aitharaju V. Enhanced ductility in in-layer glass-carbon fiber/epoxy hybrid composites produced via tailored fiber placement. Composites Part A: Applied Science and Manufacturing. 2023;168:107488. doi: 10.1016/j.compositesa.2023.107488

[43] Singh S, Kumar Gupta P. Effect of fiber orientation on mechanical properties of jute/carbon/glass hybrid composite. Materials Today: Proceedings. 2022;68:2574–80. doi: 10.1016/j.matpr.2022.09.419

[44] Özyer T, Demirci E. Evaluation of Jute-Glass Ratio Effects on the Mechanical, Thermal, and Morphological Properties of PP Hybrid Composites for Sustainable Automotive Applications. Polymers (Basel). 2025;17(24):3335. doi: 10.3390/polym17243335 41471010 PMC12736671

[45] Das SC, Paul D, Grammatikos SA, Siddiquee MAB, Papatzani S, Koralli P. Effect of stacking sequence on the performance of hybrid natural/synthetic fiber reinforced polymer composite laminates. Compos Struct. 2021;276:114525. doi: 10.1016/j.compstruct.2021.114525

[46] Dong C, Davies IJ. Optimal design for the flexural behaviour of glass and carbon fibre reinforced polymer hybrid composites. Mater Des. 2012;37:450–7. doi: 10.1016/j.matdes.2012.01.021

[47] Hamed A, Megat Ahmad MMH, Sahari B, Sapuan S. Experimental characterization of filament wound glass/epoxy and carbon/epoxy composite materials. ARPN J Eng Appl Sci. 2008;3.

[48] Dong C, Davies IJ. Flexural and tensile moduli of unidirectional hybrid epoxy composites reinforced by S-2 glass and T700S carbon fibres. Materials & Design (1980-2015). 2014;54:893–9. doi: 10.1016/j.matdes.2013.08.086

[49] Zhang J, Chaisombat K, He S, Wang CH. Hybrid composite laminates reinforced with glass/carbon woven fabrics for lightweight load bearing structures. Materials & Design (1980-2015). 2012;36:75–80. doi: 10.1016/j.matdes.2011.11.006

[50] Murugan R, Ramesh R, Padmanabhan K, Jeyaraam R, Krishna S. Experimental investigation on static mechanical properties of glass/carbon hybrid woven fabric composite laminates. Adv Mat Res. 2014;903:96–101. doi: 10.4028/www.scientific.net/AMR.903.96

[51] Mohamed N, EL-Wazery M, EL-Elamy M, Zoalfakar S. Mechanical and dynamic properties of hybrid composite laminates. In: International Conference on Aerospace Sciences and Aviation Technology, 2017. 1–23. 10.21608/asat.2017.22746

[52] Dong C. Flexural properties of symmetric carbon and glass fibre reinforced hybrid composite laminates. Composites Part C: Open Access. 2020;3:100047. doi: 10.1016/j.jcomc.2020.100047

[53] Erbayrak E, Yuncuoglu EU, Kahraman Y, Gumus BE. An Experimental and Numerical Determination on Low-Velocity Impact Response of Hybrid Composite Laminate. Iran J Sci Technol Trans Mech Eng. 2020;45(3):665–81. doi: 10.1007/s40997-020-00402-4

[54] Singh H, Singh IP, Singh S, Dhawan V, Kumar Tiwari S. A brief review of jute fibre and its composites. Mater Today Proc. 2018;5:28427–37. doi: 10.1016/j.matpr.2018.10.129

[55] Rabbi M, Islam M. Jute Fiber-Reinforced Polymer Composites, A Comprehensive Review. IJMPERD. 2020;10(3):3053–72. doi: 10.24247/ijmperdjun2020290

[56] Sanjay MR, Yogesha B. Studies on Mechanical Properties of Jute/E-Glass Fiber Reinforced Epoxy Hybrid Composites. JMMCE. 2016;04(01):15–25. doi: 10.4236/jmmce.2016.41002

[57] ASTM International. ASTM D3039/D3039M-14: Standard Test Method for Tensile Properties of Polymer Matrix Composite Materials. West Conshohocken, PA. 2014. doi: 10.1520/D3039_D3039M-14

[58] ASTM International. ASTM D790-17: Test Methods for Flexural Properties of Unreinforced and Reinforced Plastics and Electrical Insulating Materials. West Conshohocken, PA: ASTM International. 2017. doi: 10.1520/D0790-17

[59] ASTM International. ASTM D3410/D3410M-16: Test method for compressive properties of polymer matrix composite materials with unsupported gage section by shear loading. West Conshohocken, PA: ASTM International. 2016. doi: 10.1520/D3410_D3410M-16

[60] Pravin L. Tension-tension fatigue testing of pultruded carbon fibre composite profiles. Aalto University. 2016.

[61] Govindaraju R, Jagannathan S. Optimization of mechanical properties of silk fiber-reinforced polypropylene composite using Box–Behnken experimental design. Journal of Industrial Textiles. 2016;47(5):602–21. doi: 10.1177/1528083716667257

[62] ANSYS Inc. ANSYS Mechanical APDL Element Reference, Release 2020 R1. Canonsburg, PA, USA: ANSYS Inc. 2020.

[63] Ilunga M, Agarwal A. A Finite-Element-Analysis-Based Feasibility Study for Optimizing Pantograph Performance Using Aluminum Metal Matrix Composites. Processes. 2024;12(3):445. doi: 10.3390/pr12030445

[64] Arora G, Sharma H, Bhowmik P, Singh MK, Ayyappan V, Rangappa SM. Archives of Computational Methods in Engineering. 2026;33:5339–67. doi: 10.1007/s11831-025-10454-x

[65] Gopinath A, Kumar MS, Elayaperumal A. Experimental Investigations on Mechanical Properties Of Jute Fiber Reinforced Composites with Polyester and Epoxy Resin Matrices. Procedia Engineering. 2014;97:2052–63. doi: 10.1016/j.proeng.2014.12.448

