# Supplementary material for: Experimental and numerical investigation of the mechanical performance of natural–synthetic hybrid composite laminates
Source: PLoS One. 2026 Jul 17;21(7):e0354203. doi: 10.1371/journal.pone.0354203 (PMC13379099; doi:10.1371/journal.pone.0354203)
Supplement: S1 File — (DOCX) [file pone.0354203.s001.docx]

**S1 Supporting Information**

Experimental and Numerical Investigation of the Mechanical Performance of Natural–Synthetic Hybrid Composite Laminates for Potential Pressure Vessel Applications

**Table S1.** Mechanical properties of the investigated laminate configurations.

| Laminate Configuration | Tensile Strength (MPa) | Compressive Strength (MPa) | Flexural Strength (MPa) |
| --- | --- | --- | --- |
| J2/C2/J2 | 108 | 38.57 | 91 |
| J2/G2/J2 | 29 | 22 | 89 |
| C2/G2/C2 | 140 | 39 | 109 |

**Table S2.** Percentage deviation between experimental and finite element results.

| Laminate Configuration | Tensile Error (%) | Compression Error (%) | Flexural Error (%) |
| --- | --- | --- | --- |
| J2/C2/J2 | 0.86 | 0.15 | 0.19 |
| J2/G2/J2 | 1.13 | 0.46 | 0.52 |
| C2/G2/C2 | 0.04 | 0.12 | 0.57 |

**Table S3.** Orthotropic material properties used in finite element simulations.

| Material | E1 (GPa) | E2 (GPa) | G12 (GPa) | ν12 | Density (kg/m³) |
| --- | --- | --- | --- | --- | --- |
| Jute/Epoxy | 10.5 | 3.5 | 1.8 | 0.30 | 1350 |
| Glass/Epoxy | 38 | 8.5 | 4.2 | 0.28 | 1950 |
| Carbon/Epoxy | 65 | 6 | 4.5 | 0.30 | 1600 |

**Table S4.** Mesh convergence study for J2/C2/J2 laminate under compression loading.

| Element Size (mm) | Number of Elements | Number of Nodes | Predicted von Mises Stress (MPa) |
| --- | --- | --- | --- |
| 5 | 140 | 170 | 38.671 |
| 4 | 245 | 288 | 39.027 |
| 3 | 423 | 480 | 38.513 |
| 2 | 910 | 994 | 37.048 |

Experimental compressive strength: 38.571 MPa.
